# PortNet: Achieving lightweight architecture and high accuracy in lung cancer cell classification

**DOI:** 10.1016/j.heliyon.2025.e41850

**Published:** 2025-01-09

**Authors:** Kaikai Zhao, Youjiao Si, Liangchao Sun, Xiangjiao Meng

**Affiliations:** a Department of Radiation Oncology, Shandong Cancer Hospital and Institute, Shandong First Medical University and Shandong Academy of Medical Sciences, Jinan, Shandong, China; b Department of Radiology, Shandong Cancer Hospital and Institute, Shandong First Medical University and Shandong Academy of Medical Sciences, Jinan, Shandong, China

**Keywords:** Lung cancer, Pathology, Artificial intelligence-assisted bioinformatic analysis, Deep learning

## Abstract

**Background:**

As one of the cancers with the highest incidence and mortality rates worldwide, the timeliness and accuracy of cell type diagnosis in lung cancer are crucial for patients' treatment decisions. This study aims to develop a novel deep learning model to provide efficient, accurate, and cost-effective auxiliary diagnosis for the pathological types of lung cancer cells.

**Method:**

This paper introduces a model named PortNet, designed to significantly reduce the model's parameter size and achieve lightweight characteristics without compromising classification accuracy. We incorporated 1 × 1 convolutional blocks into the Depthwise Separable Convolution architecture to further decrease the model's parameter count. Additionally, the integration of the Squeeze-and-Excitation self-attention module enhances feature representation without substantially increasing the number of parameters, thereby maintaining high predictive performance.

**Result:**

Our tests demonstrated that PortNet significantly reduces the total parameter count to 2,621,827, which is over a fifth smaller compared to some mainstream CNN models, marking a substantial advancement for deployment in portable devices. We also established widely-used traditional models as benchmarks to illustrate the efficacy of PortNet. In external tests, PortNet achieved an average accuracy (ACC) of 99.89 % and Area Under the Curve (AUC) of 99.27 %. During five-fold cross-validation, PortNet maintained an average ACC of 99.51 % ± 1.50 % and F1 score of 99.50 % ± 1.51 %, showcasing its lightweight capability and exceptionally high accuracy. This presents a promising opportunity for integration into hospital systems to assist physicians in diagnosis.

**Conclusion:**

This study significantly reduces the parameter count through an innovative model structure while maintaining high accuracy and stability, demonstrating outstanding performance in lung cancer cell classification tasks. The model holds the potential to become an efficient, accurate, and cost-effective auxiliary diagnostic tool for pathological classification of lung cancer in the future.

## 1 Introduction

Lung cancer, due to its high incidence and mortality, has become one of the most severe global health issues [[1], [2], [3]]. Its impact is particularly evident in two aspects: firstly, the survival period for lung cancer patients is typically short, especially in the advanced stages of the disease where the treatment options are limited, leading to an exceedingly high mortality rate; secondly, lung cancer symptoms are often not apparent in the early stages, and by the time symptoms manifest, the disease is frequently in an advanced stage, missing the optimal treatment window and further increasing the difficulty of treatment and economic burden on patients. Moreover, the treatment process for lung cancer often comes with significant physical and mental burdens, affecting the quality of life of patients. The cellular classification of lung cancer mainly falls into two categories: Non-Small Cell Lung Cancer (NSCLC) and Small Cell Lung Cancer (SCLC) [4]. NSCLC accounts for about 85 % of all lung cancers and primarily includes three subtypes: adenocarcinoma, squamous cell carcinoma, and large cell carcinoma [5]. Adenocarcinoma is the most common subtype, often found in non-smokers and women; squamous cell carcinoma has a strong correlation with smoking; large cell carcinoma is relatively rare and has a poorer prognosis. These subtypes differ in biological characteristics, growth patterns, treatment responses, and prognosis, making their accurate identification crucial for clinical decision-making. Small Cell Lung Cancer accounts for the remaining 15 %, and despite its lower incidence, it grows rapidly and can spread extensively early on, making it more challenging to treat and leading to a grimmer prognosis than NSCLC.

Differentiating between these lung cancer cell types is vitally important for the selection of clinical treatment strategies and prognosis assessment. However, due to the morphological similarities between different subtypes of lung cancer and the disease's heterogeneity, even experienced pathologists face significant challenges in classifying lung cancer cells [6]. This process often requires meticulous histopathological examination under a microscope and may need various special stains and molecular biological tests to assist in diagnosis. Although these diagnostic methods can provide accurate typing information to some extent, they are usually labor-intensive, requiring specialized skills and high levels of concentration, thus being time-consuming and strenuous. Additionally, these techniques are generally costly, and in resource-limited areas, the high diagnostic costs can become a significant burden for patients and the healthcare system. At the same time, the acquisition and processing of lung cancer samples are challenging. The quality of samples can vary greatly due to differences in collection, transportation, storage, etc., and these variations may affect the accuracy and repeatability of pathological diagnoses. For example, certain cellular characteristics may change during the specimen preparation process, leading to misdiagnosis or missed diagnosis. Additionally, there may be discrepancies between different pathologists' diagnoses, adding uncertainty to the diagnosis and possibly requiring multiple reviews or further molecular diagnostics for confirmation. All these factors can prolong the diagnostic time for patients, increase their psychological and economic burden, and simultaneously affect the timeliness and effectiveness of treatment.

In this context, developing a rapid, accurate, and cost-effective technology to differentiate lung cancer cell types could have a profound impact on the diagnosis and treatment of lung cancer. Such technology would simplify the diagnostic process, shorten diagnostic time, reduce medical costs, and at the same time, improve the accuracy and consistency of diagnoses. For patients, this means they could receive definitive diagnostic results faster and thereby start appropriate treatment sooner, potentially improving treatment outcomes and survival rates. For the healthcare system, it would help optimize resource allocation and enhance the overall efficiency of medical services. Therefore, developing rapid diagnostic technology is of significant importance in clinical settings and is one of the hot topics in current lung cancer research.

Significant progress has been made in the study of lung cancer pathological classification using deep learning, particularly in cell morphological analysis. Various models such as Convolutional Neural Networks (CNNs), Transformers have been applied, with architectures like Inception and ResNet standing out for their image classification accuracy [[7], [8], [9], [10]]. These models, by automatically identifying and learning patterns in images, can assist doctors in faster and more accurate lung cancer typing. However, these models also have limitations. First, they generally require a large amount of labeled training data, which is particularly challenging in the medical field as high-quality medical imaging data is not readily available. Second, the interpretability issues of the models limit their application in clinical practice. It might be difficult for doctors and clinical practitioners to understand the decision-making process of the models, posing a barrier to establishing trust in the models and adopting them for medical decision-making. Finally, the training cost of the models is a significant consideration. Traditional deep learning models currently require high computational power during the training process, meaning the corresponding medical product development costs are high, posing challenges for product transformation. Future research needs to strive for model lightweighting, enhancing the interpretability of models, and reducing reliance on large amounts of labeled data.

**Fig. 1:**
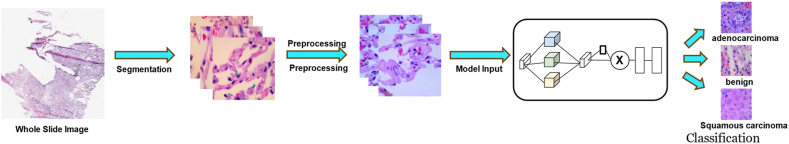
The workflow of this study. As a lightweight and highly accurate lung cancer cell classification model, PortNet's potential clinical value lies in its ability to assist pathologists in rapid screening and diagnosis, improving diagnostic efficiency, especially in resource-constrained environments. In addition, the efficiency of PortNet makes it possible to be deployed in portable devices or cloud platforms, facilitating the sharing and interoperability of diagnostic results between different medical institutions, thereby promoting the popularization of early diagnosis of lung cancer.

This study aims to develop a model that maintains high accuracy while being lightweight, for the automatic detection and classification of various pulmonary diseases. We introduce a model named PortNet. Within PortNet, we initially incorporate residual blocks composed of 1 × 1 convolutions into the Depthwise Separable Convolution (DSC) framework. This integration enhances feature expression and reduces parameter count, building upon the existing strengths of DSC. Furthermore, a Squeeze-and-Excitation self-attention module follows, designed to augment feature representation without significantly increasing parameter size, thus preserving high predictive performance. The model culminates in an average pooling layer, which expands to the appropriate size for the fully connected layer, producing predictions under a SoftMax activation function before final output. To assess our model's capability comprehensively and objectively in predicting and classifying lung diseases, we conducted external testing and five-fold cross-validation. We also compared PortNet with several deep learning models widely used by researchers, offering a holistic view of its performance. The workflow of this study is depicted in Fig. 1.

## 2 Method

### 2.1 Database

**Fig. 2:**
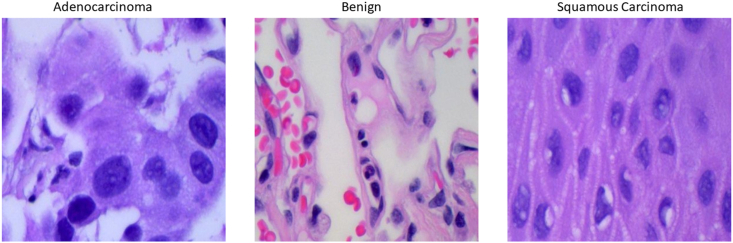
The diagram of Lung and Colon Cancer database. This figure displays representative images of different types of lung cancer cells, including adenocarcinoma, benign cells, and squamous carcinoma.

In this study, we utilized the Lung and Colon Cancer database from Kaggle (https://www.kaggle.com/datasets/biplobdey/lung-and-colon-cancer). This dataset comprises 25,000 histopathological images, distributed equally across five classes, each containing 5000 images. Each image measures 768 × 768 pixels and is presented in JPEG format. These images originate from HIPAA-compliant and validated sources, encompassing a total of 750 lung tissue images (250 benign lung tissues, 250 lung adenocarcinomas, and 250 lung squamous cell carcinomas) and 500 colon tissue images (250 benign colon tissues and 250 colon adenocarcinomas). The dataset was expanded to 25,000 images using the Augmentor package. Specifically, we perform a variety of transformations on the image, such as random rotation, horizontal flipping, and brightness adjustment, to improve the robustness and generalization ability of the model. For the purpose of this research, we specifically selected three categories pertaining to pulmonary diseases: Lung benign tissue, Lung adenocarcinoma, and Lung squamous cell carcinoma, as our training data. This selection is pivotal for enabling the model to predict and classify various cellular forms of lung cancer. Fig. 2 presents schematic representations of cell scanning images from various classifications within the Lung and Colon Cancer database. This visualization aids in illustrating the diverse cell types and morphologies encountered in the dataset, providing a foundational understanding of the imaging characteristics pivotal for our analysis.

### 2.2 Data preprocessing

Before feeding the images into the model, a series of preprocessing steps were undertaken. Initially, mean filtering was applied to remove surface noise from the images, thereby mitigating its potential impact on model predictions. Subsequently, we employed Gaussian high-pass filters to extract and subsequently integrate edge details back into the original images. This dual approach not only counters the edge information loss due to mean filtering but also accentuates edge details, facilitating the model's ability to recognize cellular shapes. Furthermore, histogram equalization was performed in the brightness space to enhance image clarity and readability, thereby bolstering the model's recognition capabilities. Additionally, for the purposes of external testing and more rigorous validation, 1250 images from the original dataset were segregated, with an equal split into validation and test sets.

### 2.3 Model construction

#### 2.3.1 PortNet

In the preliminary experimental validation of this study, it was observed that most models demonstrated superior performance in distinguishing and classifying different types of pulmonary tumor cells, achieving accuracies around 99 %. However, many of these models possess substantial parameter sizes, posing challenges for deployment on portable medical devices with limited computational power. Hence, this research aims to develop a model that maintains high classification accuracy while significantly reducing parameter size and achieving lightweight architecture. To this end, we introduce the PortNet model, a fusion model based on DSC, residual blocks, and Squeeze-and-Excitation self-attention mechanism.

Depthwise separable convolution, proposed by Howard et al. [11] in 2017 as part of the MobileNet architecture, includes two stages: Depthwise Convolution and Pointwise Convolution. Initially, Depthwise Convolution applies separate convolutions for each input channel, followed by Pointwise Convolution that combines the output of the depthwise convolution to mix feature channels. This convolution technique drastically reduces computational and parameter requirements, making the model more lightweight and suitable for mobile or resource-constrained settings. This innovation has been particularly valuable in mobile applications and edge computing, enabling rapid and efficient image and video analysis. It also holds significant implications in medical image recognition, marking a milestone in the application of deep learning models, especially in portable medical devices [[12], [13], [14]].

Residual blocks, introduced by He et al. [15] in 2015 as part of the ResNet architecture, play a crucial role in deep learning. These blocks incorporate skip connections, allowing gradients to flow directly through the network. This design alleviates the vanishing gradient problem, making training of deeper networks more feasible. Residual blocks have been instrumental in image recognition, classification, and detection tasks, advancing the field of deep learning in medicine, with increasing adoption in medical studies in recent years [[16], [17], [18]].

Squeeze-and-Excitation self-attention, conceived by Hu et al. [19] in 2017, is an attention mechanism in neural network architectures. It explicitly models inter-channel dependencies to enhance the performance of convolutional neural networks. The mechanism includes two steps: generating global spatial information for each channel through global average pooling, followed by fully connected layers that capture inter-channel dependencies. Its key feature is the network's ability to adaptively recalibrate channel-wise feature responses, emphasizing useful features while suppressing less important ones. This simple yet effective approach has shown significant performance improvements across various network architectures and is extensively used in scenarios where performance enhancement is sought without significant increase in complexity [[20], [21], [22]].

**Fig. 3:**
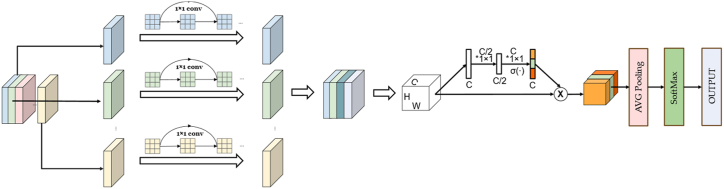
The construction of PortNet. This figure illustrates the structural design of PortNet, highlighting its convolutional layers, feature extraction modules, and classification pathway. The network begins with a series of 1 × 1 convolution layers to reduce dimensionality and capture essential features, followed by a main processing block that combines feature maps. The final layers consist of average pooling, softmax activation, and output classification, enabling accurate and efficient lung cancer cell categorization.

The newly developed PortNet model innovatively reduces its parameter count by integrating 1 × 1 convolution-based residual blocks intoDSC. This approach effectively minimizes the model's parameter size, thereby achieving further lightweight capabilities beyond its existing framework. In addition, the PortNet model incorporates a Squeeze-and-Excitation (SE) self-attention mechanism post-hoc, which enhances the representation of features without significantly inflating the number of parameters. This integration is crucial as it preserves the model's high predictive performance while maintaining a compact architecture. The structural configuration of the PortNet model is delineated in Fig. 3, providing a visual representation of its innovative design.

#### 2.3.2 Baseline models

To objectively evaluate the proposed PortNet, we constructed several models widely utilized by researchers for comparison (ResNet, Xception, Inception, DenseNet, and VGG). These models are extensively applied and have validated their exceptional performance and generalization capability across different tasks, offering direction for our model's development. Xception, proposed by Chollet et al., in 2016 [23], is an evolution of the Inception model. It enhances performance and efficiency by replacing the standard convolution in the Inception module with DSC. DSC first applies a depthwise convolution along the spatial dimension, followed by a pointwise convolution. This approach reduces the number of model parameters and improves computational efficiency. The model has excelled in various image recognition tasks and has been widely applied in medical research [24,25]. Inception, introduced by the Google team's Szegedy et al., in 2014 [26], is notable for its complex deep architecture, featuring "Inception modules." These modules allow the network to capture information at different scales by applying various sizes of convolutional filters parallelly within the same network layer. For instance, an Inception module may simultaneously have 1 × 1, 3 × 3, and 5 × 5 convolutions, along with a 3 × 3 max pooling, with all outputs concatenated. This design enables the network to adaptively learn the convolutional kernel sizes best suited for a specific problem. DenseNet, proposed by Huang et al., in 2017 [27], is characterized by its dense inter-layer connections. In DenseNet, each layer is directly connected to all preceding layers, allowing the network to more effectively utilize feature information, enhancing feature propagation and reuse, reducing parameter count, and decreasing the risk of overfitting. The VGG network, a widely used deep learning convolutional neural network architecture, was introduced by Simonyan et al., in 2014 [28]. It builds a deep architecture by repetitively using small-sized convolutional kernels (3 × 3) and max pooling layers (2 × 2), effectively extracting image features. By comparing PortNet with these established models, the study not only demonstrates the exceptional performance of PortNet in recognizing pulmonary diseases but also identifies areas for improvement and future development directions.

### 2.4 Model evaluation

To objectively evaluate the proposed PortNet model, we used Accuracy (ACC), Precision (PRE), Recall (REC), F1 Score (F1), and Area Under the ROC Curve (AUC) as performance metrics. These are calculated from True Positives (TP), True Negatives (TN), False Positives (FP), and False Negatives (FN), representing four distinct outcomes in model predictions compared to actual labels:

● True Positives (TP): Cases where the model correctly predicts positive.
● True Negatives (TN): Cases where the model correctly predicts negative.
● False Positives (FP): Cases where the model incorrectly predicts positive.
● False Negatives (FN): Cases where the model incorrectly predicts negative.

ACC is the ratio of correctly classified instances to the total number of instances, representing the model's overall performance. The formula for ACC is:

(1) $$ACC=\frac{TP+TN}{TP+FP+TN+FN}$$

PRE measures the accuracy of positive predictions, calculated as:

(2) $$PRE=\frac{TP}{TP+FP}$$

REC indicates the model's ability to capture positive instances, calculated as:

(3) $$REC=\frac{TP}{TP+FN}$$

The F1 Score is the weighted average of precision and recall, giving equal importance to both:

(4) $$F1=\frac{2\times PRE\times REC}{PRE+REC}$$

The ROC curve is a graphical evaluation tool showing the performance of classification models across all possible thresholds. It plots True Positive Rate (TPR or Recall) against False Positive Rate (FPR). TPR is the model's ability to correctly identify positive samples, while FPR is the frequency of incorrectly identifying negative samples as positive. An ideal ROC curve approaches the top left corner, indicating low FPR and high TPR. The AUC represents the area under the ROC curve, quantifying the overall performance of a classifier, with values ranging between 0 and 1. An AUC of 1 signifies perfect classification, 0.5 indicates ineffective classification (unable to distinguish between positive and negative classes), and less than 0.5 suggests that classification is worse than random guessing.

## 3 Result

### 3.1 Experimental set up

In this study, we employed a gradient threshold method to adjust the parameters of all models, ensuring the objectivity and efficacy of our evaluations. Through extensive experimentation and testing, optimal epochs and learning rates were determined to ensure full model convergence without the occurrence of overfitting. This approach also confirms that the models did not settle into local optima. Additionally, we set the batch size to 32, a decision dictated by the constraints of available computational resources. This careful parameter tuning enabled efficient model training within the limits of our hardware capabilities.

The experimental procedures in this research were conducted using a system running Windows 11 Professional. Python 3.10.9 served as the programming environment, facilitating the development and validation of the model's architecture. For software libraries, we utilized Pytorch 2.0.1+cu117, Scikit-learn 0.0.post1, and Scipy 1.10.0, along with other essential mathematical libraries, to support the computational requirements of the study. The hardware configuration included an Intel Core i7 10750H processor, which operates at a base frequency of 2.6 GHz and can turbo boost up to 5 GHz. This processor features 6 cores and 12 threads, providing robust computational power. Additionally, our system was equipped with an NVIDIA GeForce GTX 1080Ti graphics card, offering 8 GB of memory and a 128-bit memory bus width, thereby enhancing the processing capabilities for complex model computations.

### 3.2 Results of 5-fold cross-validation

**Fig. 4:**
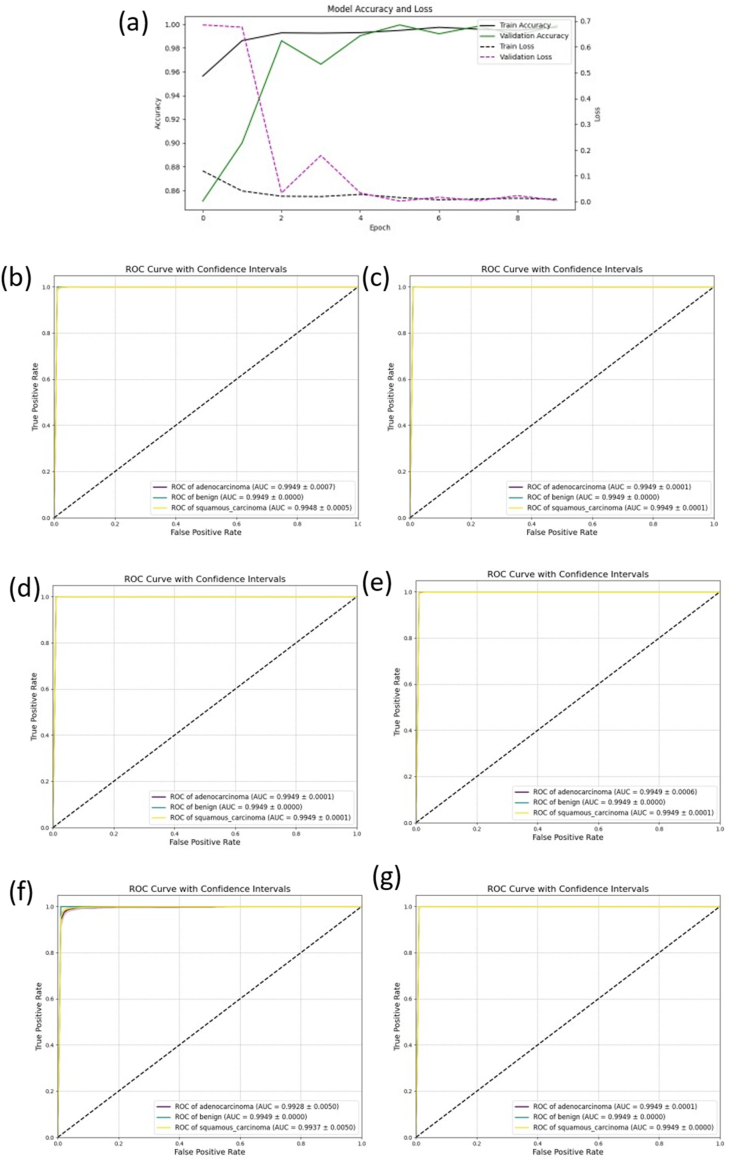
The result of five-fold cross-validation (a) The training flow of PortNet (b) The ROC curve of PortNet (c)–(g) The ROC curve of DenseNet, Inception, ResNet, VGG and Xception.

To assess the performance and stability of the newly developed PortNet model, we employed five-fold cross-validation on the training dataset. The dataset was segmented into five equal parts, with each segment serving as a test set in a rotational training cycle. The training was executed for 10 epochs at a learning rate of 0.00001, ensuring model convergence without overfitting, as illustrated in Fig. 4(a).

PortNet demonstrated exceptional efficacy, achieving an average ACC and F1 score of 99.51 % ± 1.50 % and 99.50 % ± 1.51 %, respectively, with their 95 % confidence intervals. These results exceeded most comparative models and exhibited superior stability with minimal prediction fluctuations. Notably, PortNet's parameter count is significantly lower, almost one-tenth of that of the comparative models. The outcomes of the five-fold cross-validation across all models are detailed in Table 1. The ROC curve for PortNet is displayed in Fig. 4(b), and the ROC curves for the comparative models are shown in Fig. 4(c)–(g).

**Table 1:** The results of 5-fold cross validation.

Models	Mean Accuracy	Mean Precision	Mean Recall	Mean F1
**PortNet**	**99.51 %** ± **1.50 %**	**99.60 %** ± **1.39 %**	**99.51 %** ± **1.50 %**	**99.50 %** ± **1.51**
ResNet	98.68 % ± 0.78 %	98.72 % ± 0.74 %	98.68 % ± 0.78 %	98.67 % ± 0.78 %
Xception	99.87 % ± 0.01 %	99.87 % ± 0.01 %	99.87 % ± 0.01 %	99.87 % ± 0.01 %
Inception	99.17 % ± 1.00 %	99.21 % ± 0.92 %	99.17 % ± 1.00 %	99.17 % ± 1.00 %
Dense	95.31 % ± 5.39 %	96.40 % ± 3.71 %	95.31 % ± 5.39 %	95.14 % ± 5.68 %
VGG	96.63 % ± 1.78 %	96.94 % ± 1.37 %	96.63 % ± 1.78 %	96.62 % ± 1.81 %

### 3.3 Results of the independent test set

**Fig. 5:**
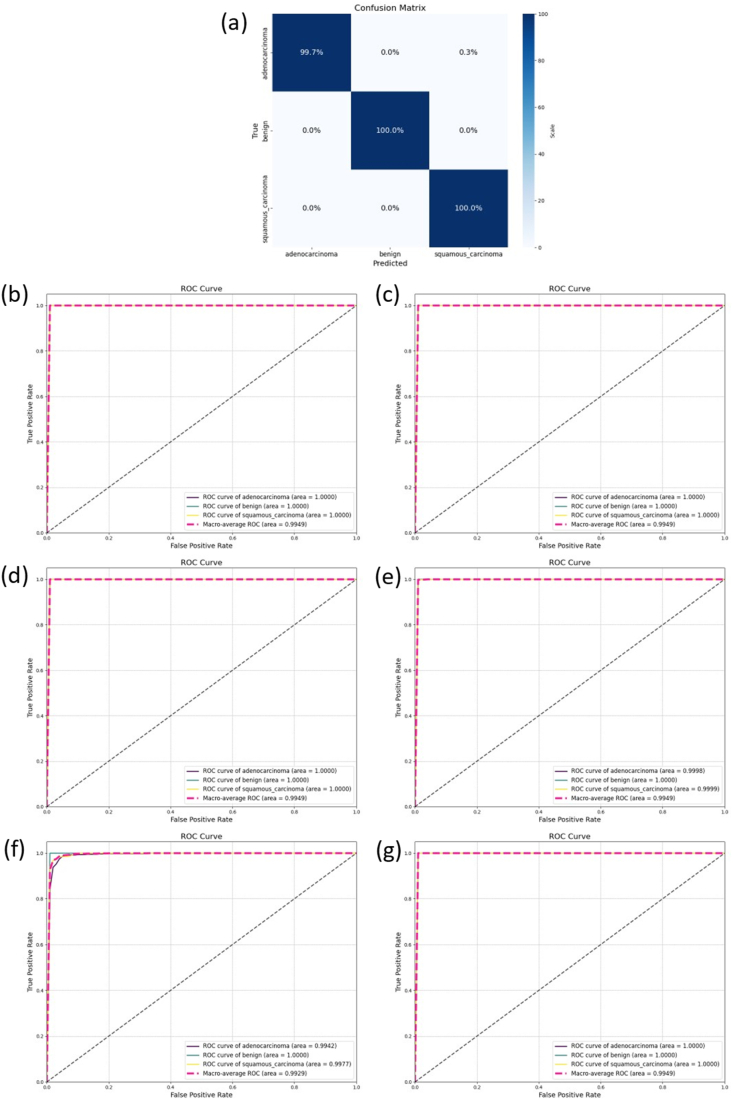
The result of external validation (a) The confusion matrix of PortNet (b) The ROC curve of PortNet (c)–(g) The ROC curve of DenseNet, Inception, ResNet, VGG and Xception.

To ascertain PortNet's predictive efficiency on unseen data, external testing was executed using the test set. The model was configured for 10 epochs at a learning rate of 0.00001. Optimal epochs and learning rates were similarly identified for the comparative models, ensuring unbiased and effective outcomes. In external testing, PortNet's exemplary performance was evident, with its confusion matrix presented in Fig. 5(a). It achieved an average ACC and F1 score of 99.89 %, and an AUC of 99.49 %, nearing perfection in predictive capability.

PortNet's parameter count is 2,621,827, substantially less than the comparison model with the lowest parameter count, DenseNet, which has 7,563,843 parameters. Other models compared in the study have parameter counts around 20,000,000, almost tenfold higher than PortNet. This marked reduction in parameters significantly enhances PortNet's portability, suggesting its potential for integration into portable devices for aiding physicians in categorizing lung cell cancer types. The table depicting the performance of each model in external tests is shown as Table 2, and the ROC curves for the external tests are depicted in Fig. 5(b)–(g).

**Table 2:** The results of the independent test set.

Models	Mean Accuracy	Mean Precision	Mean Recall	Mean F1	Total Params
**PortNet**	**99.89 %**	**99.89 %**	**99.89 %**	**99.89 %**	**2,621,827**
ResNet	98.40 %	98.40 %	98.40 %	98.40 %	24,638,339
Xception	100.00 %	100.00 %	100.00 %	100.00 %	21,912,107
Inception	99.63 %	99.63 %	99.63 %	99.63 %	22,853,411
Dense	94.81 %	94.81 %	94.81 %	94.81 %	7,563,843
VGG	99.73 %	99.73 %	99.73 %	99.73 %	14,978,883

### 3.4 Model visualization and clinical interpretability

**Fig. 6:**
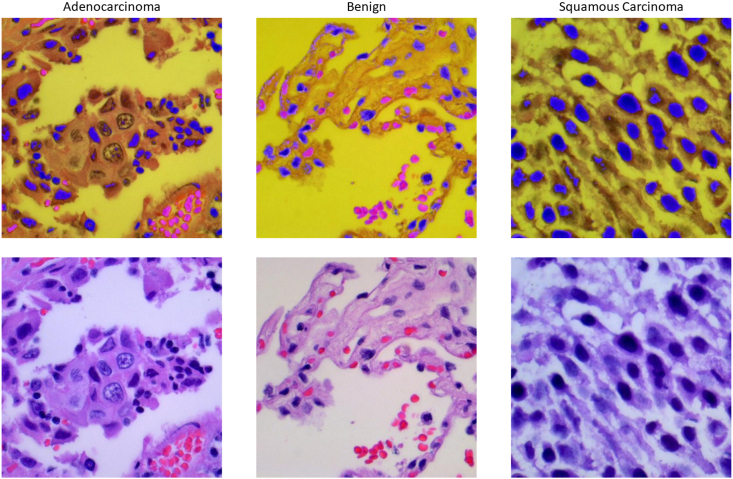
The heat map of PortNet's prediction results. The top row presents examples of adenocarcinoma, benign, and squamous carcinoma cells with color overlays highlighting areas of interest. The bottom row shows the corresponding original images. The heat maps illustrate PortNet's focus areas within each image, providing insights into the model's classification decisions and enhancing interpretability of the results.

The interpretability of the model is directly linked to the acceptance level by both physicians and patients. Therefore, it is essential to conduct an interpretability analysis of the PortNet model proposed in this study. We elucidate the model's predictive results through the generation of visual class activation heatmaps. This method focuses on the regions emphasized by the model during prediction to ascertain whether the model is correctly analyzing and making judgments. Fig. 6 displays the visual class activation heatmaps of PortNet for different classifications. From the figure, it is evident that the model accurately identifies cell regions and their shapes, and bases its judgments on these cellular morphologies. This approach aligns with the standards for distinguishing different cell types in pulmonary tumors, thereby enabling effective and highly accurate decisions.

## 4 Discussion

In the realm of identifying and classifying various lung tumor cells, most existing models exhibit exemplary performance, achieving an accuracy rate of approximately 99 %. This demonstrates significant potential for application in medical systems to alleviate the workload of healthcare professionals. However, a major limitation of many models is their substantial parameter size, which poses challenges for integration into portable medical devices with limited computational power. Addressing this issue, the current study introduces a novel model, PortNet, which significantly reduces the parameter size without compromising classification accuracy, thereby facilitating its deployment in lightweight medical devices. PortNet innovatively combines depthwise separable convolutions with 1 × 1 convolutions into a residual block. This design strategy aims to further reduce the parameter size beyond the capabilities of traditional Depthwise Separable Convolution. Additionally, the model incorporates a Squeeze-and-Excitation self-attention module subsequent to this block. This module enhances feature representation without substantially increasing the number of parameters, thereby maintaining high predictive performance.

Empirical testing reveals that PortNet dramatically reduces the total parameter count to 2,621,827. This figure represents a near ten-fold decrease compared to some mainstream Convolutional Neural Network (CNN) models, marking a significant advancement for deployment on portable devices. Despite its reduced parameter size, PortNet exhibits exceptional predictive performance and stability. In a five-fold cross-validation setup, PortNet achieved an average ACC and F1-score, along with their 95 % confidence intervals, of 99.51 % ± 1.50 % and 99.50 % ± 1.51 % respectively. These results are close to 100 %, indicating outstanding predictive capability. Additionally, the model demonstrates minimal variation across different training batches, signifying high stability. In conclusion, the PortNet model holds promise for deployment in clinical settings. It can assist physicians in diagnosing various types of cellular cancers and selecting appropriate treatment methods, thereby reducing the burden on clinical practitioners.

The remarkable performance of the PortNet model in the precise identification and classification of various lung tumor cells suggests its vast potential for widespread application in lung cancer pathological classification. As a lightweight and highly accurate lung cancer cell classification model, PortNet's potential clinical value lies in its ability to assist pathologists in rapid screening and diagnosis, improving diagnostic efficiency, especially in resource-constrained environments. In addition, the efficiency of PortNet makes it possible to be deployed in portable devices or cloud platforms, facilitating the sharing and interoperability of diagnostic results between different medical institutions, thereby promoting the popularization of early diagnosis of lung cancer. This model not only accurately and efficiently identifies different types of tumor cells but also performs detailed subtyping of tumors, which has significant clinical importance in guiding personalized treatment plans and predicting therapeutic effects. As lung cancer treatment strategies continuously evolve, the ability to rapidly and accurately classify cells will directly impact the selection of treatment plans and optimization of therapeutic effects. The application of PortNet can enhance the efficiency and accuracy of this process, allowing doctors to provide more personalized treatment recommendations based on more precise diagnostic information. On the other hand, the lightweight architecture of PortNet makes it suitable for use in resource-limited environments, such as mobile health applications and remote medical platforms. With the development of cloud computing and IoT technology, PortNet can be easily integrated into smart medical devices for real-time tumor monitoring and classification. In remote areas lacking professional pathologists, this lightweight model is expected to realize rapid and low-cost diagnosis of lung cancer.

Despite the superior performance of the PortNet model in lung cancer cell classification, this study still has some limitations. Firstly, the dataset used in this study comes from a public database, which, while providing certain conveniences and feasibility, also limits the comprehensive assessment of the model's generalizability. In future research, we need to validate the model in more medical centers and use multi-source, multi-center datasets to test and enhance the generalizability of the PortNet model, ensuring it maintains efficient diagnostic performance across different regions, devices, and diverse patient populations. Secondly, although PortNet has achieved significant results in reducing parameters, there is still room for optimization. Future research needs to explore more advanced network structures and learning algorithms to further reduce model parameters and improve computational efficiency and model stability. Finally, the actual application of the PortNet model is still in the preliminary stage, and the corresponding deployment system has not yet been developed. To make the model effective in real medical environments, future work needs to include developing user-friendly interfaces, integrating the model into medical imaging devices, and ensuring compatibility and stability of the model across different hardware and software environments.

## 5 Conclusion

This study aims to develop a novel deep learning model to address the limitations in parameter size and computational resource demands of existing lung cancer cell classification models, thereby making them more suitable for portable medical devices. By utilizing depthwise separable convolutions combined with 1 × 1 convolutions in residual blocks, and incorporating a Squeeze-and-Excitation self-attention module, the proposed PortNet model significantly reduces parameter count while maintaining a high level of recognition accuracy. The results of this study suggest that the PortNet model has considerable potential for widespread clinical application, especially in scenarios requiring portable diagnostic tools. It can assist pathologists in efficiently and accurately diagnosing various types of lung cancer cells at a lower development cost. Despite this, we recognize the limitations of this study, including the need for further validation of the model's generalizability and the lack of a deployment system, which will be addressed in future work.

## CRediT authorship contribution statement

**Kaikai Zhao:** Writing – original draft. **Youjiao Si:** Formal analysis. **Liangchao Sun:** Software, Methodology. **Xiangjiao Meng:** Writing – review & editing.

## Ethical declaration

Since the data analyzed in this paper are sourced from publicly available databases, this study does not require an ethical review. Public databases typically contain anonymized data that do not pose risks to individual privacy, thus exempting this research from the usual ethical approval processes associated with studies involving human subjects.

## Data availability statement

The original contributions presented in the study can be directed to the corresponding authors.

## Funding

This work was supported by the 10.13039/501100001809National Natural Science Foundation of China (grant numbers 81972796, 81972863).

## Declaration of Competing Interest

The authors declare the following financial interests/personal relationships which may be considered as potential competing interests: Xiangjiao Meng reports financial support was provided by 10.13039/501100015507Shandong First Medical University Cancer Hospital. Xiangjiao Meng reports a relationship with Shandong First Medical University Cancer Hospital that includes: employment. If there are other authors, they declare that they have no known competing financial interests or personal relationships that could have appeared to influence the work reported in this paper.

## Abbreviations

NSCLC: Non-Small Cell Lung Cancer
SCLC: Small Cell Lung Cancer
CNNs: Convolutional Neural Networks
DSC: Depthwise Separable Convolution
SE: Squeeze-and-Excitation
AUC: Area Under the ROC Curve
ACC: Accuracy
PRE: Precision
REC: Recall
F1: F1 Score
TP: True Positives
TN: True Negatives
FP: False Positives
FN: False Negatives
TPR: True Positive Rate
FPR: False Positive Rate
